# IGF2BP3 mediates the mRNA degradation of NF1 to promote triple‐negative breast cancer progression via an m6A‐dependent manner

**DOI:** 10.1002/ctm2.1427

**Published:** 2023-09-24

**Authors:** Xu Zhang, Liang Shi, Han‐Dong Sun, Zi‐Wen Wang, Feng Xu, Ji‐Fu Wei, Qiang Ding

**Affiliations:** ^1^ Jiangsu Breast Disease Center The First Affiliated Hospital with Nanjing Medical University Nanjing China; ^2^ Department of Pharmacy Jiangsu Cancer Hospital The Affiliated Cancer Hospital of Nanjing Medical University Jiangsu Institute of Cancer Research Nanjing China

**Keywords:** IGF2BP3, m6A, NF1, TET3, TNBC

## Abstract

**Background:**

N6‐methyladenosine (m6A) is an abundant reversible modification in eukaryotic mRNAs. Emerging evidences indicate that m6A modification plays a vital role in tumourigenesis. As a crucial reader of m6A, IGF2BP3 usually mediates the stabilisation of mRNAs via an m6A‐dependent manner. But the underlying mechanism of IGF2BP3 in the tumourigenesis of triple‐negative breast cancer (TNBC) is unclear.

**Methods:**

TCGA cohorts were analysed for IGF2BP3 expression and IGF2BP3 promoter methylation levels in different breast cancer subtypes. Colony formation, flow cytometry assays and subcutaneous xenograft were performed to identify the phenotype of IGF2BP3 in TNBC. RNA/RNA immunoprecipitation (RIP)/methylated RNA immunoprecipitation (MeRIP) sequencing and luciferase assays were used to certify the target of IGF2BP3 in TNBC cells.

**Results:**

IGF2BP3 was highly expressed in TNBC cell lines and tissues. TET3‐mediated IGF2BP3 promoter hypomethylation led to the upregulation of IGF2BP3. Knocking down IGF2BP3 markedly reduced the proliferation of TNBC in vitro and in vivo. Intersection co‐assays revealed that IGF2BP3 decreased neurofibromin 1 (NF1) stabilisation via an m6A‐dependent manner. NF1 knockdown could rescue the phenotypes of IGF2BP3 knockdown cells partially.

**Conclusion:**

TET3‐mediated IGF2BP3 accelerated the proliferation of TNBC by destabilising NF1 mRNA via an m6A‐dependent manner. This suggests that IGF2BP3 could be a potential therapeutic target for TNBC.

## Graphical abstract

- TET3‐mediated promoter hypomethylation leads to upregulation of IGF2BP3 expression in TNBC.
- The TET3/IGF2BP3/NF1 axis in the regulation of TNBC proliferation and apoptosis.
- IGF2BP3 could decrease the stability of the targeted gene expression as an m6A reader.

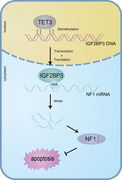

## 1 INTRODUCTION

Breast cancer (BC) is the most common tumour and remains the leading cause of cancer‐related deaths among women worldwide, posing a severe threat to the health and lives of women.^1^, ^2^ BC is divided into four subtypes: luminal A, luminal B, HER2‐enriched and triple‐negative breast cancer (TNBC).^3^ Different BC subtypes show varied characteristics concerning biological properties, therapy strategies and clinical prognosis.^4^ Among them, TNBC is characterised by higher grade, larger tumour size and poor survival, accounting for about 20% of all BC patients.^5^, ^6^ More than 70% of metastatic TNBC patients are not alive 5 years after diagnosis and show the worst outcomes compared to other subtypes.^7^ Therefore, it is vital to delve into the underlying molecular mechanisms of TNBC and develop novel treatment strategies.

At present, epigenetic modification currently plays a crucial role in the occurrence and development of different tumours.^8^ N6‐methylladenosine (m6A) is the most common modification of eukaryotic mRNAs^9^, ^10^ and influences mRNA splicing, localisation, export, translation, decay and stability.^11^, ^12^, ^13^ m6A modification is a reversible and dynamic process,^14^ introduced by methyltransferases (‘writers’) and demethyltransferases (‘erasers’).^15^, ^16^ In addition, m6A modification is functionally executed by ‘readers’, which are primarily defined as ‘readers’ that mediate pre‐mRNA processing, mRNA stability, degradation and translation processes.^17^, ^18^, ^19^, ^20^

IGF2BP3, also known as IMP3, was first discovered because of its high expression in pancreatic cancer.^21^ Then, IGF2BP3 was quickly interpreted to be the primary overexpressed among multiple tumours, including lung cancer,^22^ ovarian cancer,^23^ liver cancer,^24^ osteosarcoma,^25^ bladder cancer^26^ and BC,^27^ and the abnormal upregulation showed a potential role in the tumourigenesis. Previous studies have confirmed that IGF2BP3 acts in various critical biological pathways and is involved in various critical cellular functions in BC. For instance, IGF2BP3 facilitated PDZ‐binding motif activation by stabilising Wnt family member 5B (WNT5B) mRNA in BC.^28^ It promoted chemoresistance by regulating association of tennis professionals‐binding cassette subfamily G (ABCG) expression in BC.^29^ Furthermore, IGF2BP3 promoted stem‐like properties by regulating SLUG in TNBC.^30^ IGF2BP3 also promoted the metastasis through the destabilisation of progesterone receptors in BC.^31^ However, IGF2BP3 participated in the tumour progression as an RNA‐binding protein and did not involve m6A‐related mechanism in these studies.

Herein, we explored the function of IGF2BP3 in TNBC and explored the deeper m6A‐related mechanisms by which IGF2BP3 regulates the proliferation and apoptosis of TNBC. First, we demonstrated that IGF2BP3 was upregulated and correlated with poor prognosis in TNBC compared to other BC subtypes. We then found that TET3‐mediated hypomethylation of IGF2BP3 promoter led to the upregulation of IGF2BP3 in TNBC. In addition, we found that IGF2BP3 participated in the regulation of TNBC cell proliferation and apoptosis in vivo and in vitro. mRNA sequencing (mRNA‐seq), RNA immunoprecipitation (RIP) sequencing (RIP‐seq) and methylated RNA immunoprecipitation (MeRIP) sequencing (MeRIP‐seq) demonstrated the direct target of IGF2BP3 as neurofibromin 1 (NF1) in TNBC. Therefore, IGF2BP3 acts as an oncogene in TNBC by regulating NF1 mRNA stabilisation through an m6A‐dependent manner and is a potential target to treat TNBC.

## 2 MATERIALS AND METHODS

### 2.1 Cell culture

The human BC cell lines MCF‐7, BT474, ZR‐75‐1, SK‐BR‐3, MDA‐MB‐453, HCC‐1806, MDA‐MB‐231, and BT549 and breast epithelial cell line MCF‐10A were purchased from ATCC. Among them, MCF‐7 and ZR‐75‐1 are considered luminal subtypes. BT474 and SK‐BR‐3 represent the HER2‐positive subtype, and MDA‐MB‐453, MDA‐MB‐231, HCC‐1806 and BT549 belong to the TNBC subtypes.

The cells were maintained in Dulbecco's modified Eagle's medium (Wisent); in addition, HCC‐1806 cells were maintained in Roswell Park Memorial Institute‐1640 (Wisent), which were all supplemented with 10% foetal bovine serum and 1% penicillin–streptomycin and incubated at 37°C in a humid environment with 5% CO_2_.

### 2.2 Lentivirus and siRNA transfection

Lentiviruses were produced for knockdown and overexpression of IGF2BP3. The MDA‐MB‐231 and HCC‐1806 cells were transfected with IGF2BP3 knockdown (termed as shIGF2BP3‐1 and shIGF2BP3‐2) lentivirus and matched negative control (named as shRNA‐NC), IGF2BP3 overexpression (named as IGF2BP3) lentivirus and matched negative control (termed as Vector) (Obio Technology). We finally selected the stable cell lines with 3 μg/mL puromycin. Mechanistic studies used shIGF2BP3‐1 structure with a better knockdown level of IGF2BP3 expression named shIGF2BP3.

The MDA‐MB‐231 and HCC‐1806 cells were infected with TET1‐siRNA, TET2‐siRNA, TET3‐siRNA, NF1‐siRNA (GenePharma) and non‐silencing control. The IGF2BP3‐negative control and knockdown cells MDA‐MB‐231 and HCC‐1806 were infected with NF1‐siRNA and negative control vectors (shRNA‐NC + Vector, shIGF2BP3 + Vector, shRNA‐NC + siNF1, shIGF2BP3 + siNF1) (GenePharma). The sequences of lentivirus and siRNAs are shown in Table S1.

### 2.3 qRT‐PCR

Quantitative real time polymerase chain reaction (qRT‐PCR) was conducted as previously described.^32^ The specific PCR primers were designed for Invitrogen Trading (Shanghai) and are shown in Table S2.

### 2.4 Western blot

Western blotting was conducted as previously described.^32^ The antibodies were as follows: anti‐rabbit IGF2BP3 (14642‐1‐AP, Proteintech), TET3 (DF13335, Affinity), caspase 3 (19677‐1‐AP, Proteintech), caspase 9 (10380‐1‐AP, Proteintech), NF1 (27249‐1‐AP, Proteintech) and anti‐mouse β‐actin (66009‐1‐Ig, Proteintech).

### 2.5 CCK‐8 assay

Cell proliferation was detected by the cell counting kit‐8 (CCK‐8) kit (Vazyme, Chian) as previously described.^33^ In a word, cells were plated in a 96‐well plate at 2000 cells/well. The absorbance was measured by OD450 for 2 h after adding 10% CCK‐8 solution.

### 2.6 Colony formation assay

The colony formation assay was conducted as previously described.^34^ Cells were maintained in six‐well plates at 5 × 10^3^ cells/well.

### 2.7 EdU assay

Following the manufacturer's instructions, EdU assay was performed using an EdU Cell Proliferation Kit (C0075, Beyotime). In brief, cells were maintained in a 96‐well plate at 2 × 10^4^ cells/well and incubated with 50 μM EdU per well. Then, the cells were fixed with 4% paraformaldehyde and stained with 1× click reaction buffer and 1× Hoechst 33342 solution. Finally, the cells were counted under the Zeiss fluorescence photomicroscope.

### 2.8 Flow cytometry analysis

The cell apoptosis rate was probed by an annexin annexin V‐APC (V‐APC)/7‐Aminoactinomycin D (7‐AAD) apoptosis kit (MultiSciences Biotech). Cells in different treatment groups were cultured for 24 h, and 3 × 10^5^ cells (including culture supernatant cells) were obtained. Then, cells were resuspended in 300 μL of 1× binding buffer, and then 10 μL of 7‐AAD and 5 μL of annexin V‐APC were added. After vertexing gently and incubating away from light, samples were analysed by flow cytometry (FCM) in the BDFACSCalibur system.

### 2.9 Animal models

BALB/c nude mice (4–6 weeks) were randomly divided into four groups. Stable shRNA‐NC, shIGF2BP3, shRNA‐NC + siNF1 and shIGF2BP3 + siNF1 MDA‐MB‐231 cells were injected into each group of mice (1 × 10^7^ cells/mouse). In addition, tumour volumes were recorded every 4 days. Animal experiments were carried out following the ethical standards of experimental animal institutions approved by the Animal Management Committee of Nanjing Medical University.

### 2.10 Methylation‐specific PCR

The cells were extracted following the instructions of QIAmp DNA Mini Kit (Qiagen). After diluting 1 μL of DNA sample by 50 times, the DNA was converted to unmethylated cytosine bisulfite according to the instructions of the Epi Tect Bisulfite Kit (Qiagen). After the bisulphite modification, the DNA was eluted and purified and methylation‐specific PCR (MS‐PCR) was then performed. Methylation‐specific primer sequences are shown in Table S2.

### 2.11 ChIP‐qPCR

The EZ‐Magna ChIP Kit (17‐10086, Merck) was performed for ChIP. Briefly, MDA‐MB‐231 and HCC‐1806 cells were fixed with formaldehyde. Next, the cells were obtained and treated with lysis buffer, followed by sonication. The supernatants were then obtained and mixed with protein A/G magnetic beads and indicated antibodies. After washing and eluting, the DNA was obtained and quantified by qRT‐PCR.

### 2.12 mRNA high‐throughput sequencing

According to the manual's protocol, TRIzol reagent (Takara) was used to isolate total RNA from stable IGF2BP3 knockdown or control cells (MDA‐MB‐231). Three micrograms of RNAs was selected and performed to construct the library with the RNA Sample Pre Kit. The filtered sequences were then compared to the reference genome (hg38). The library construction and nextgeneration sequencing (NGS) were conducted by Beijing Allwegene (Beijing).

### 2.13 RIP sequencing

The RIP assay was performed by RNA IP lysis buffer (Millipore). MDA‐MB‐231 cell lysate was mixed with anti‐rabbit IGF2BP3 (14642‐1‐AP, Proteintech) or immunoglobulin G (IgG) at 4°C for more than 8 h. The RNA–protein complexes were adsorbed by protein A/G magnetic beads, RNA purification was performed, and RNA was obtained and quantified by qRT‐PCR. The library was sequenced using the PE 150 sequencing strategy on the Illumina Hiseq 4000 platform of Beijing Allwegene.

### 2.14 MeRIP sequencing

MeRIP assay was performed by Magna MeRIP m6A Kit (17‐10499, Millipore). Total RNAs were obtained from MDA‐MB‐231 cells. Purified RNAs were decomposed by Fragmentation Buffer. After fragmentation, cell lysate was mixed with m6A antibody (ab208577, Abcam). The RNA fragments were first ligated at the 3′ end before the chemical reaction. They were then reverse transcribed before the 3′ adapters were ligated to the final cDNA. During reverse transcription, only fragments containing internal m6A sites were incorporated, which could be further detected by high‐throughput sequencing. The library construction and NGS were conducted by Beijing Allwegene.

### 2.15 mRNA stability analysis

IGF2BP3 knockdown and overexpression cells were plated into six‐well plates and then treated with 5 μg/mL actinomycin D (ActD) at 0, 2, 4 and 6 h. Total RNAs were isolated to detect the relative NF1 mRNA levels.

### 2.16 Luciferase assay

The MDA‐MB‐231 and HCC‐1806 cells were maintained in 24‐well plates and incubated until 70% confluence. Cells were infected with negative pGL3 reporter and luciferase vector (NF1‐A, NF1‐B, NF1‐C, NF1‐D, NF1‐E, NF1‐B‐mut, NF1‐C‐mut). The mutant luciferase vector was missing m6A motif sequence. After 2 days, firefly and renilla luciferase activities were tested (Promega). The primers for vectors are shown in Table S3.

### 2.17 TCGA databases and associated analysis tools

TCGA‐BRCA (https://cancergenome.nih.gov) contains 142 TNBC and 695 non‐TNBC cases. The overall survival (OS) of TNBC patients was obtained by KM plotter (http://www.kmplot.com). The expression of eight m6A ‘readers’ was analysed, summarised and visualised by the R software package ‘complexheatmap’. The ‘Ggplot2’ package was applied to gene ontology (GO) pathway enrichment analysis and the ‘clusterProfiler’ package was applied to gene set enrichment analysis (GSEA) of these differentially expressed genes (DEGs).

### 2.18 Statistical analysis

All statistical analyses were performed using the SPSS 19.0 software. All experiments were performed in triplicate unless otherwise stated. Linear correlation analysis was used to evaluate the correlation between IGF2BP3 and NF1. For all the continuous variables, Student's *t*‐test and two‐way ANOVA were performed to compare statistical significance between groups. ^*^ *p* < .05 was considered statistically significant.

## 3 RESULTS

### 3.1 IGF2BP3 was upregulated in TNBC and associated with a poorer prognosis

**FIGURE 1:**
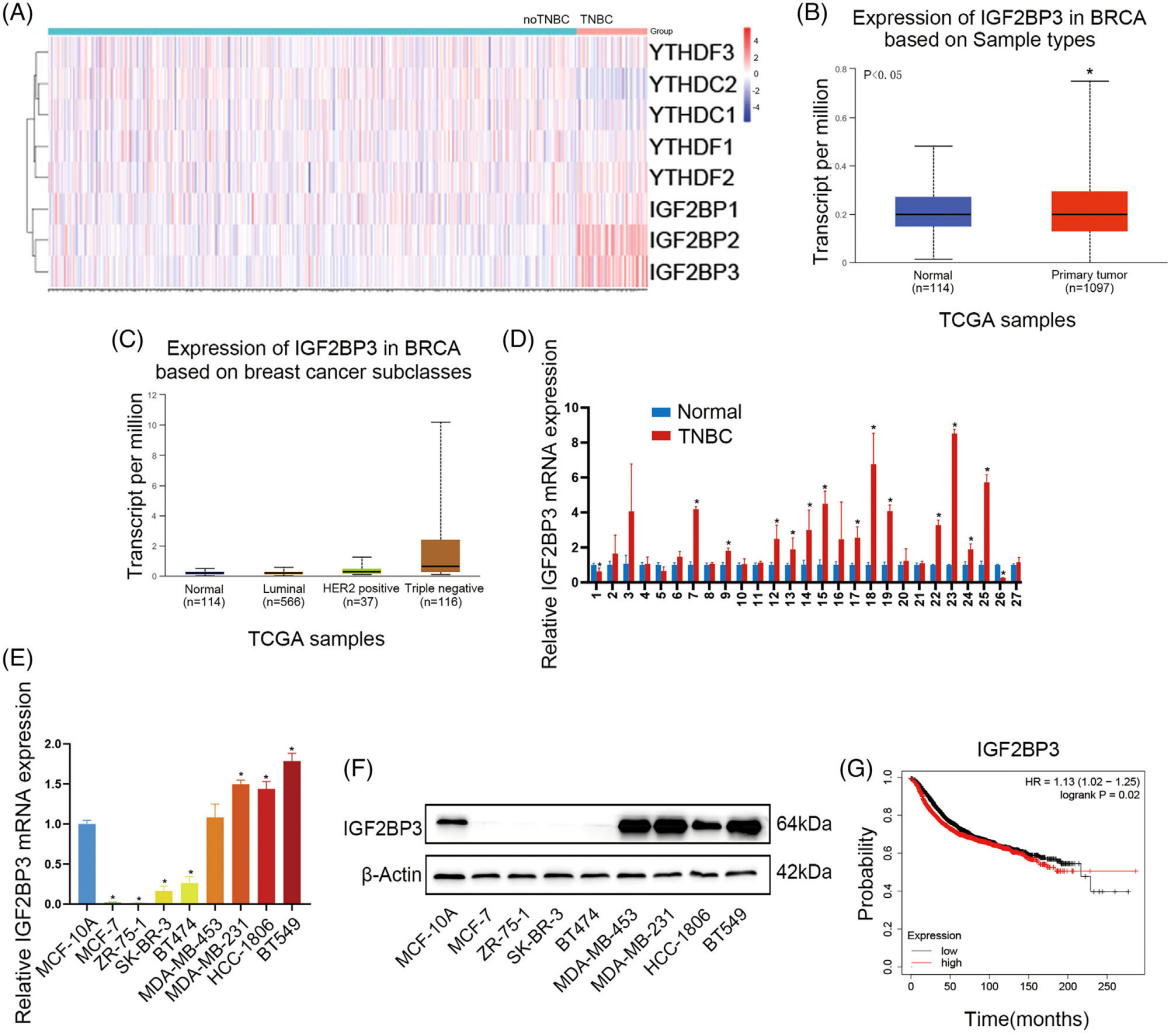
IGF2BP3 was upregulated in triple‐negative breast cancer (TNBC) and correlated with a poor prognosis. (A) Heatmap showing RNA differential expression of eight N6‐methyladenosine (m6A) ‘readers’ between TNBC and non‐TNBC. (B) Expression of IGF2BP3 in normal tissue (*n* = 114) and breast cancer (*n* = 1097) from TCGA dataset. (C) Expression of IGF2BP3 in different molecular subtypes of breast cancer. (D) IGF2BP3 mRNA expression in 27 pairs of TNBC and adjacent normal tissues. The relative quantification was calculated by the 2^−ΔΔCt^ method. (E) mRNA expression of IGF2BP3 in different cell lines. (F) Protein expression of IGF2BP3 in different cell lines. The relative quantification was calculated by the 2^−ΔCt^ method. (G) Kaplan–Meier analysis of the overall survival of TNBC patients. Data are shown as the mean ± SEM; ^*^ *p* < .05.

To identify distinctive molecular signatures among different BC subtypes, we performed a comprehensive analysis of TCGA datasets between TNBC and non‐TNBC for gene expression differences. Among the eight m6A ‘readers’, IGF2BP3 and IGF2BP2 mRNAs were obviously higher in TNBC samples than in non‐TNBC samples (Figure 1A). Moreover, IGF2BP3 mRNAs were also upregulated in BC tissues compared to normal tissues (Figure 1B), especially in TNBC tissues (Figure 1C). We further proved that the expression of IGF2BP3 in TNBC tissues was higher than that in adjacent normal tissues (Figure 1D). IGF2BP3 expression in BC cell lines was also substantiated by qRT‐PCR (Figure 1E) and western blotting (Figure 1F). Among the BC cells, IGF2BP3 was highly expressed in TNBC cell lines (BT‐549, MDA‐MB‐231, HCC‐1806, MDA‐MB‐453) compared to non‐TNBC cell lines (BT‐474, MCF‐7, SK‐BR‐3, ZR‐75‐1) at both the mRNA and protein levels. Kaplan–Meier survival curves of TNBC indicated a compelling correlation between highly expressed IGF2BP3 and poor OS in TNBC patients (^*^ *p* = .02) (Figure 1G), and these results underscore that IGF2BP3 could be a prognostic marker in TNBC.

### 3.2 The promoter of IGF2BP3 is hypomethylated in TNBC

**FIGURE 2:**
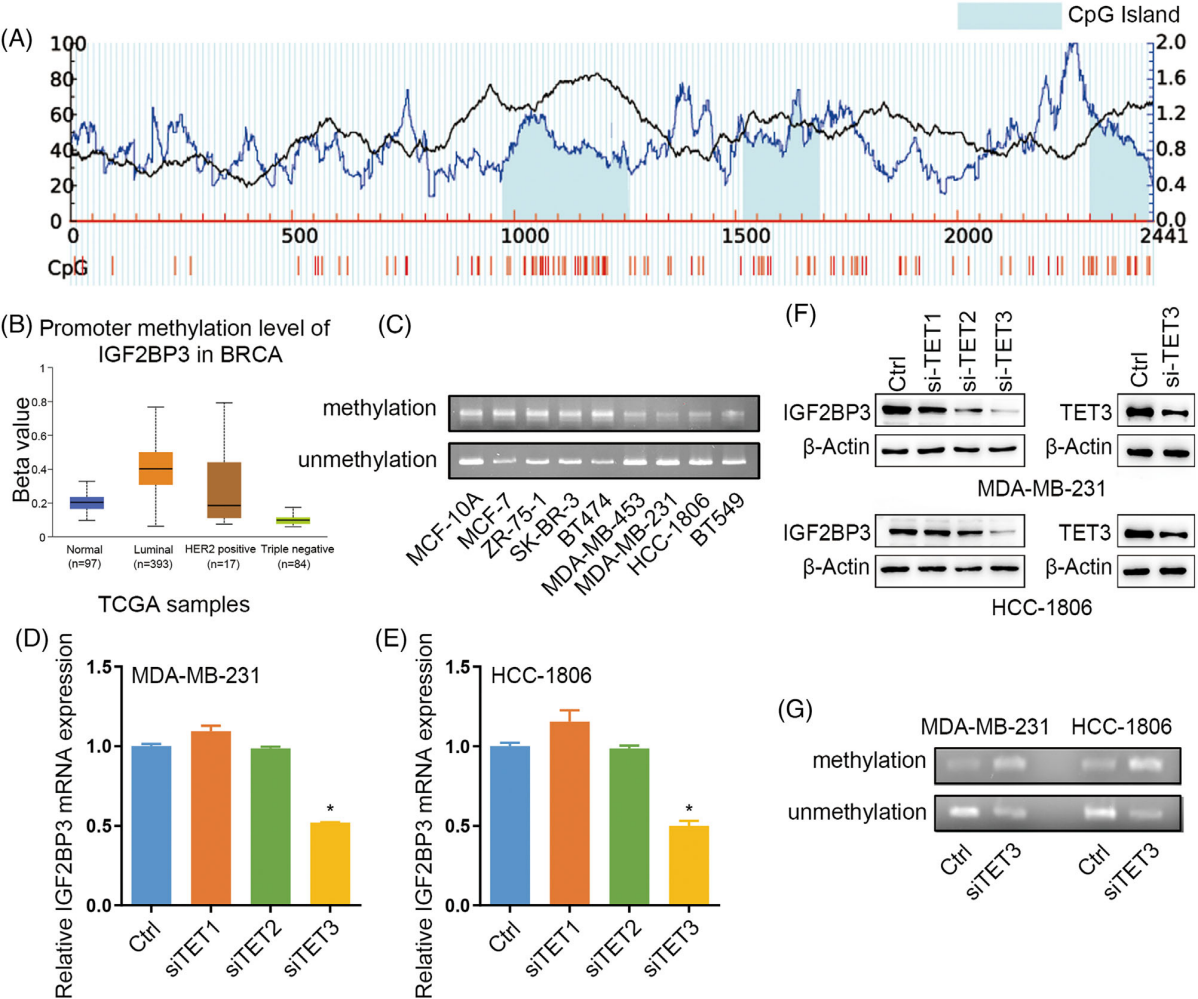
The promoter of IGF2BP3 was hypomethylated in triple‐negative breast cancer (TNBC). (A) Schematic representation of the CpG islands in the IGF2BP3 promoter. The red region is the input sequence; the blue region is CpG islands. (B) Promoter methylation level of IGF2BP3 in different molecular subtypes of breast cancer tissues from TCGA dataset. (C) Methylation‐specific PCR of the CpG island of the IGF2BP3 promoter region in different breast cancer cell lines and matched normal breast cell line. (D and E) qRT‐PCR was used to confirm IGF2BP3 expression at the mRNA level. (F) Western blotting was used to confirm the IGF2BP3 and TET3 expression at the protein level. Data are shown as the mean ± SEM; ^*^ *p* < .05. (G) Methylation‐specific PCR of the CpG island of the IGF2BP3 promoter region in TET3 knockdown MDA‐MB‐231 and HCC‐1806 cells.

IGF2BP3 expression is mediated by its promoter's DNA methylation status and transcriptional activity.^35^ Moreover, we explored whether the upregulation of IGF2BP3 was related to its promoter's methylation status in TNBC. The distinct CpG islands in the IGF2BP3 promoter are shown in Figure 2A. Notably, IGF2BP3 promoter methylation level was downregulated in TNBC tissues compared with that in non‐TNBC tissues (Figure 2B). Moreover, to delve into the transcriptional activity of IGF2BP3 gene in the different subtypes of BC, MS‐PCR analysis revealed that IGF2BP3 methylation levels in TNBC cells were substantially lower compared to those in non‐TNBC cells (Figure 2C).

DNA methylation attaches a methyl group to the carbon 5 of cytosine to create 5‐methylcytosine (5mC). This enzymatic modification is facilitated by DNA methyltransferase (DNMT) family.^36^ TET enzymes oxidise 5mC into 5‐hydroxymethylcytosine, potentially leading to DNA demethylation.^37^ To explore the potential roles of DNA demethylase in regulating IGF2BP3 promoter methylation levels in TNBC, we knocked down DNMT1/3A/3B and TET1/2/3 in MDA‐MB‐231 and HCC‐1806 cell lines using specific their siRNAs (Figure S1a–f). We found that only knockdown of TET3, and not DNMT1/3A/3B and TET1/2, resulted in a decrease in IGF2BP3 expression (Figures 2D–F and S1g–i). Figure S1j also show that IGF2BP3 was positively correlated with TET3 in 27 TNBC patient tissues in our hospital. In addition, ChIP‐qPCR experiments were performed in MDA‐MB‐231 and HCC‐1806 cell lines to demonstrate that TET3 could bind to the IGF2BP3 promoter region (Figure S1k,l). MSP analysis indicated that knockdown of TET3 increased partly the methylation level of IGF2BP3 promoter ​(Figure 2G). These results indicated that highly expressed IGF2BP3 was related to the hypomethylation of its promoter in TNBC and that TET3 led to IGF2BP3 promoter demethylation.

### 3.3 Knockdown of IGF2BP3 inhibited the proliferation and promoted the apoptosis of TNBC in vivo and in vitro

**FIGURE 3:**
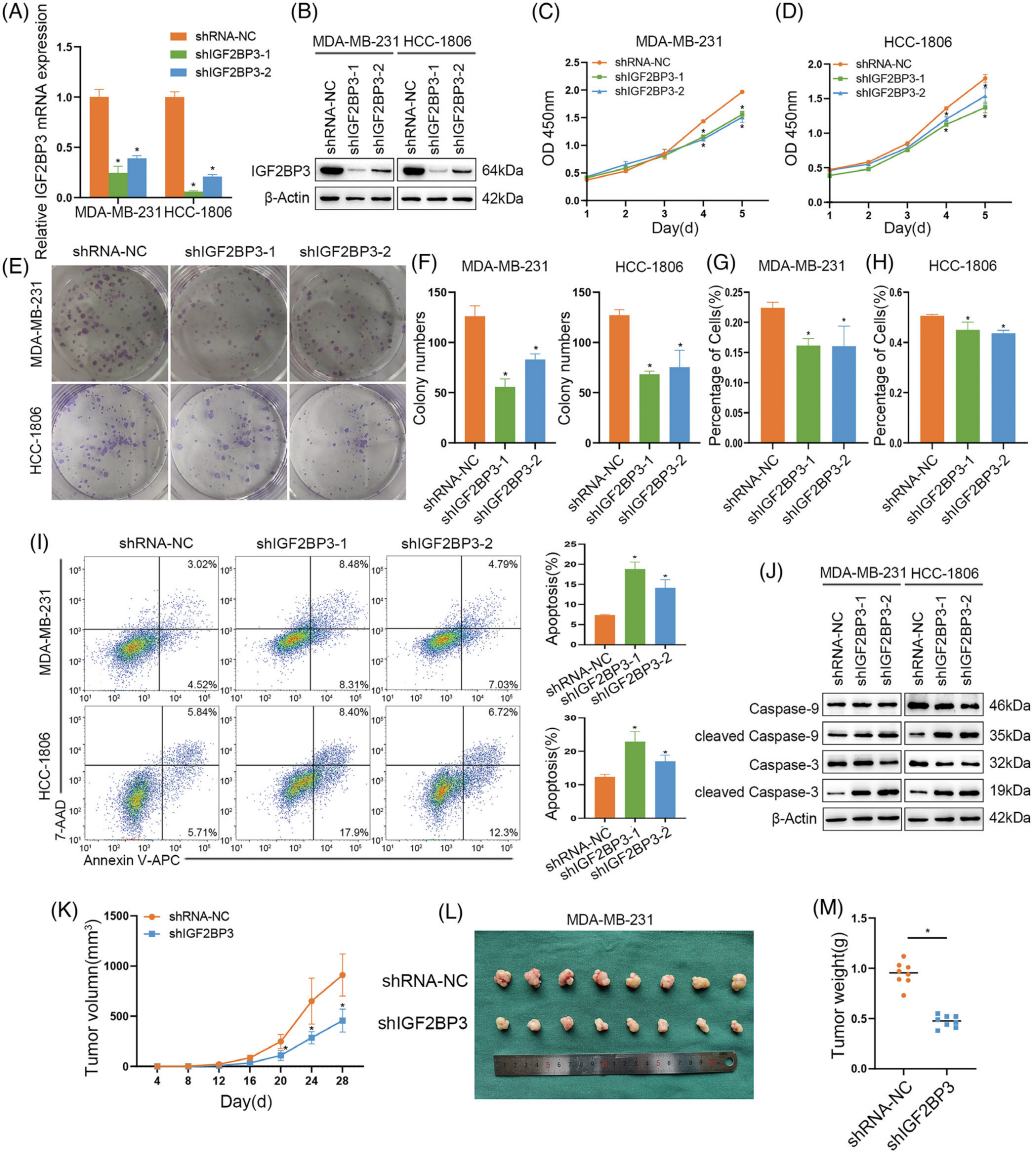
Knockdown of IGF2BP3 inhibited the proliferation and promoted the apoptosis of triple‐negative breast cancer (TNBC) in vivo and in vitro. (A and B) MDA‐MB‐231 and HCC‐1806 cell lines were transfected with lentivirus to knockdown IGF2BP3 expression (shIGF2BP3‐1, shIGF2BP3‐2). qRT‐PCR (A) and western blotting (B) were applied to verify the transfection efficiency. (C–H) CCK‐8, colony formation and EdU assays were performed in MDA‐MB‐231 and HCC‐1806 cell lines. (I and J) Flow cytometry assay and western blot were used to confirm the apoptosis analysis induced by the knockdown of IGF2BP3. (K–M) Tumour volume and weight in IGF2BP3 knockdown MDA‐MB‐231 cells compared with control at 4 weeks. Data are shown as the mean ± SEM; ^*^ *p* < .05.

To investigate the effect of IGF2BP3 in TNBC cells, MDA‐MB‐231 and HCC‐1806 cells were stably infected with IGF2BP3 knockdown and control lentiviruses. The knockdown cells were designated as shIGF2BP3‐1 and shIGF2BP3‐2, and the corresponding control was labelled as shRNA‐NC. The transfection efficiency of IGF2BP3 was validated through qRT‐PCR and western blot (Figure 3A,B). The CCK‐8, colony formation and EdU assays revealed that IGF2BP3 knockdown decreased the cell proliferation (Figures 3C–H and Figure S2a,b). Moreover, the flow cytometry analysis indicated that both early and late apoptotic cells were increased significantly upon knockdown of IGF2BP3 in MDA‐MB‐231 and HCC‐1806 cells (Figure 3I). Downregulation of IGF2BP3 increased the expression of cleaved‐caspase 3 and 9, which were responsible for morphological and biochemical changes in apoptosis (Figure 3J). In xenograft models, the tumour volume increased more slowly in the IGF2BP3 knockdown group than in the control (Figure 3K). Four weeks later, tumour volume and weights in the IGF2BP3 knockdown group were both lower than those in the control (Figure 3L,M), which indicates that knockdown of IGF2BP3 inhibited tumour proliferation in vivo.

### 3.4 IGF2BP3 overexpression stimulated the proliferation and inhibited the apoptosis of TNBC in vitro

MDA‐MB‐231 and HCC‐1806 cells were stably infected with IGF2BP3 overexpression and control lentiviruses and named as IGF2BP3 and Vector, respectively. We confirmed the IGF2BP3 expression using qRT‐PCR and western blotting (Figure S3a,b). The proliferation ability of these infected cells was examined using CCK‐8, colony formation and EdU assays. IGF2BP3 overexpression increased the cell proliferation in MDA‐MB‐231 and HCC‐1806 cells (Figure S3c–h). Moreover, the flow cytometry analysis indicated that early and late apoptotic cells significantly decreased upon overexpression of IGF2BP3 in MDA‐MB‐231 and HCC‐1806 cells (Figure S3i). Upregulation of IGF2BP3 decreased the expression of cleaved‐caspase 3 and 9 (Figure S3j).

### 3.5 Analysis of IGF2BP3 targets in TNBC

**FIGURE 4:**
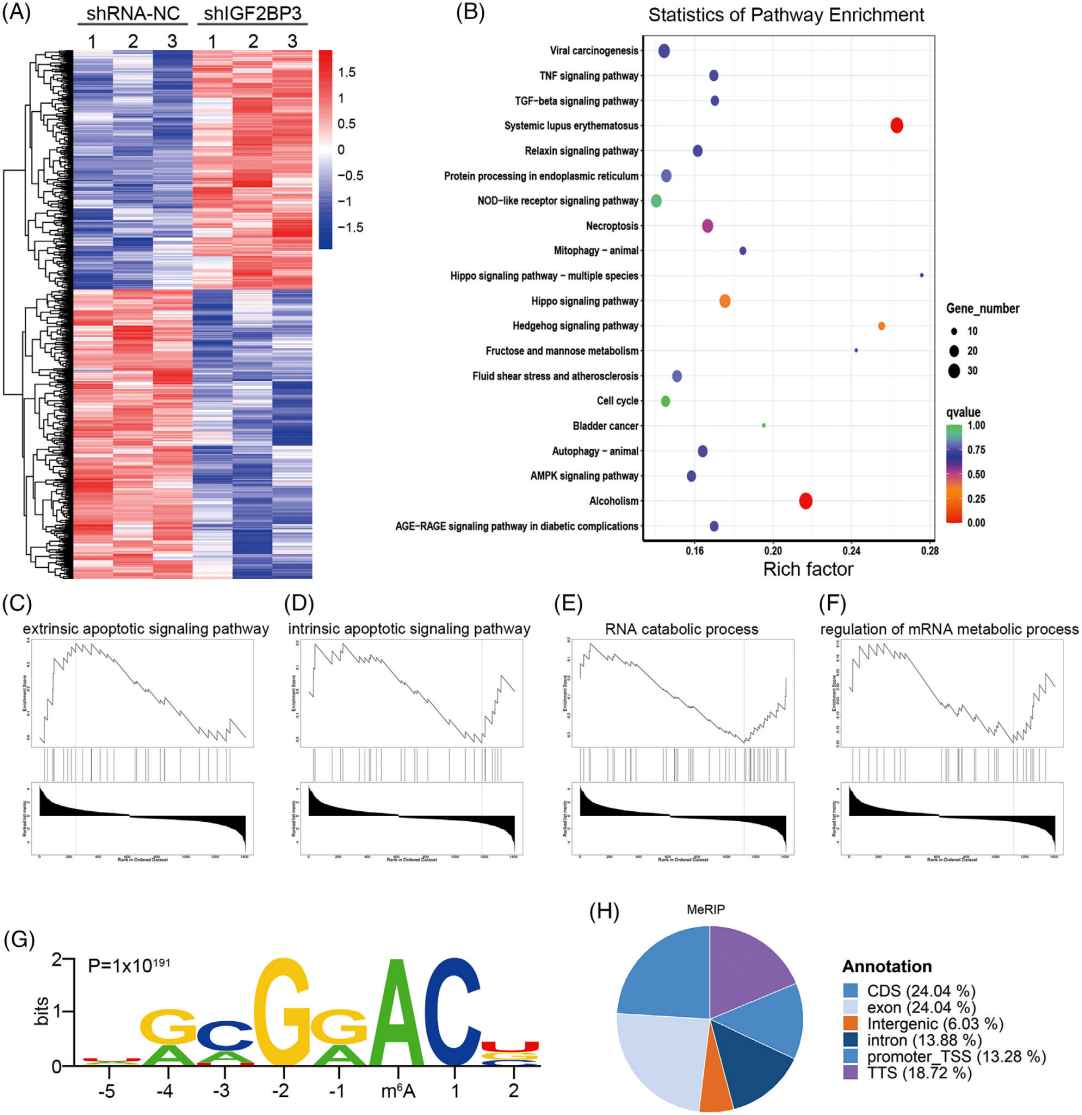
Identification of the IGF2BP3 targets in triple‐negative breast cancer (TNBC). (A) Heatmap of differentially expressed genes (DEGs) performed by RNA sequencing. (B) GO enrichment analysis of DEGs. (C–F) GSEA plots show the pathways of IGF2BP3‐enriched DEGs. (G) N6‐methyladenosine (m6A) motif detection by DREME motif analysis and m6A sequencing results. (H) Percentage of different RNA species modified by m6A.

To reveal the potential mechanisms of IGF2BP3 in TNBC, we first conducted RNA‐seq analysis of MDA‐MB‐231 cells with IGF2BP3 knockdown and control. IGF2BP3 knockdown resulted in 655 upregulated genes and 862 downregulated genes (Figure 4A). GO analysis indicated that the DEGs regulated by IGF2BP3 were related to the cell cycle, autophagy, transforming growth factor‐beta and tumour necrosis factor signalling pathway (Figure 4B). GSEA also indicated that DEGs of IGF2BP3 were related to extrinsic apoptotic and intrinsic signalling pathway, RNA catabolic process and regulation of mRNA metabolic process (Figure 4C–F), suggesting that IGF2BP3 could play an oncogenic role in TNBC. IGF2BP3 is known as an m6A reader that functions by binding and regulating m6A‐methylated mRNAs.^38^ Thus, we applied MeRIP‐seq and RIP‐seq in MDA‐MB‐231 cells. MeRIP‐seq analysis unveiled 26 904 m6A peaks corresponding to 10 939 genes. These m6A peaks were appropriately characterised by the m6A motifs (*p* = 1 × 10^−191^), which were mainly enriched in CDS regions (Figure 4G,H).

### 3.6 NF1 was an m6A target of IGF2BP3 in TNBC

**FIGURE 5:**
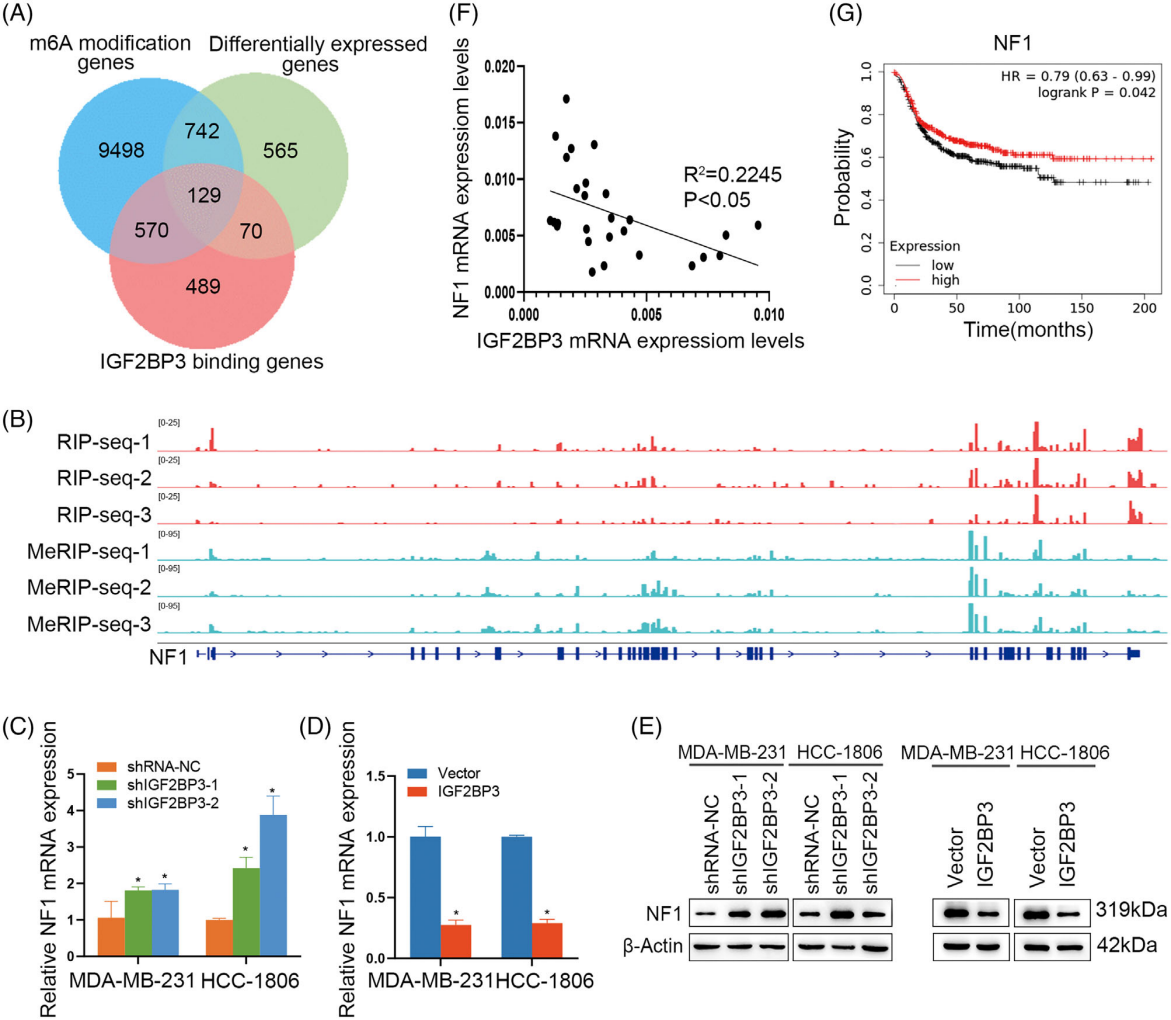
Neurofibromin 1 (NF1) was an N6‐methyladenosine (m6A) target of IGF2BP3 in triple‐negative breast cancer (TNBC). (A) Overlapping analysis of genes identified by m6A sequencing, RIP sequencing and RNA sequencing. (B) Distribution of m6A peaks and IGF2BP3‐binding peaks in transcripts. (C–E) Expression of NF1 was increased or decreased following IGF2BP3 knockdown or overexpression in MDA‐MB‐231 and HCC‐1806 cells at mRNA (C and D) and protein levels (E). Data are shown as the mean ± SEM; ^*^ *p* < .05. (F) Correlation analysis between IGF2BP3 and NF1 mRNA expression in TNBC tissues (*n* = 27). (G) Kaplan–Meier analysis of overall survival of TNBC patients.

By overlapping these genes from RIP‐seq, RNA‐seq and MeRIP‐seq, we obtained 129 genes (Table S4) bound by IGF2BP3 and modified with m6A (Figure 5A). Based on the KEGG pathway analysis through DAVID (https://david.ncifcrf.gov), three candidate genes (NF1, BCL2 and GSK3B) were identified, which were all related to cell apoptosis. Then, the impact of IGF2BP3 on these three candidate genes was evaluated by qRT‐PCR. In contrast, BCL2 and GSK3B showed almost no variations in IGF2BP3 knockdown or overexpression cells (Figure S4a–d). As a result, we selected NF1, which encodes neurofibromin and acts as a tumour suppressor with Ras‐GAP activity^39^ and affects the proliferation and apoptosis of the tumour. We found that most IGF2BP3‐binding sites in NF1 fit well with the m6A‐modified sites (Figure 5B). Then, qRT‐PCR and western blot analyses indicated that NF1 might be the potential target of IGF2BP3 (Figure 5C–E). Figure 5F indicates that IGF2BP3 was negatively associated with NF1 expression in 27 TNBC patient tissues in our hospital. Kaplan–Meier survival curves of TNBC indicated that low NF1 expression was correlated with unfavourable OS in TNBC patients (^*^ *p* = .042) (Figure 5G).

### 3.7 IGF2BP3 regulated NF1 mRNA expression via m6A‐dependent manner

**FIGURE 6:**
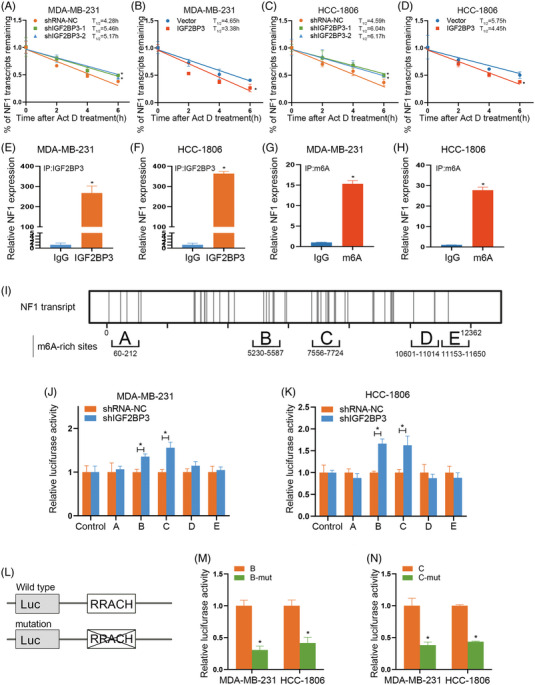
IGF2BP3 regulated neurofibromin 1 (NF1) mRNA expression via N6‐methyladenosine (m6A)‐dependent manner. (A–D) MDA‐MB‐231 and HCC‐1806 cells were treated with 5 μg/mL actinomycin D (ActD) for 0, 2, 4 and 6 h, followed by qRT‐PCR and western blot analysis. (E–H) MDA‐MB‐231 and HCC‐1806 cell lysates were immunoprecipitated with IGF2BP3 or m6A antibody and control immunoglobulin G (IgG) to detect NF1 mRNA expression. (I) Schematic diagram of regions in the NF1 mRNA. (J and K) The luciferase activity for the reporter involving NF1‐A, B, C, D, E and pGL3 was transfected by knocking down IGF2BP3 in MDA‐MB‐231 and HCC‐1806 cells. (L) Schematic diagram of mutation regions in the NF1 mRNA. (M and N) The luciferase activity for the reporter involving NF1‐B, B‐mut, C and C‐mut was transfected in MDA‐MB‐231 and HCC‐1806 cells. Data are shown as the mean ± SEM; ^*^ *p* < .05.

To investigate how IGF2BP3 mediates NF1 expression, IGF2BP3 knockdown and overexpression MDA‐MB‐231 cells were incubated with 5 μg/mL ActD at various time points. We found that downregulation of IGF2BP3 expression increased the half‐life of NF1 mRNA (Figure 6A). On the contrary, IGF2BP3 overexpression decreased the half‐life of NF1 mRNA (Figure 6B). Similar results were confirmed in HCC‐1806 cell lines (Figure 6C,D). These results confirmed that IGF2BP3 could reduce NF1 expression by regulating the mRNA stability. Moreover, RIP assay was performed in MDA‐MB‐231 and HCC‐1806 cells. The results indicated that NF1 mRNA was tested in the input and IGF2BP3 group but not in the IgG (Figure 6E,F). In addition, MeRIP assay results showed that NF1 could be bound to m6A sites (Figure 6G,H). To determine whether IGF2BP3 regulates the NF1 expression via m6A‐dependent manner, we conducted a dual‐luciferase assay in MDA‐MB‐231 and HCC‐1806 cells to identify the m6A sites that were necessary for the IGF2BP3 binding to NF1 mRNA. Sites A–E containing m6A‐rich sites were designed. Furthermore, pGL3 was designed as the negative control (Figure 6I). The activity of the luciferase reporters carrying NF1‐B and C was decreased by the knockdown of IGF2BP3. Additionally, the NF1‐A, D and E did not respond to IGF2BP3 (Figure 6J,K). Moreover, the activity of luciferase reporters carrying NF1‐B‐mut and C‐mut decreased compared with that of the control group (Figure 6L–N). Generally, these results showed that IGF2BP3 could directly connected to the NF1‐B and C sites of NF1 mRNA to decrease NF1 expression via m6A‐dependent manner, indicating that the specific m6A modification sites were located at the 5291, 5450 and 7587 base.

### 3.8 NF1 reversed the proliferation inhibition and apoptosis promotion induced by IGF2BP3 knockdown

**FIGURE 7:**
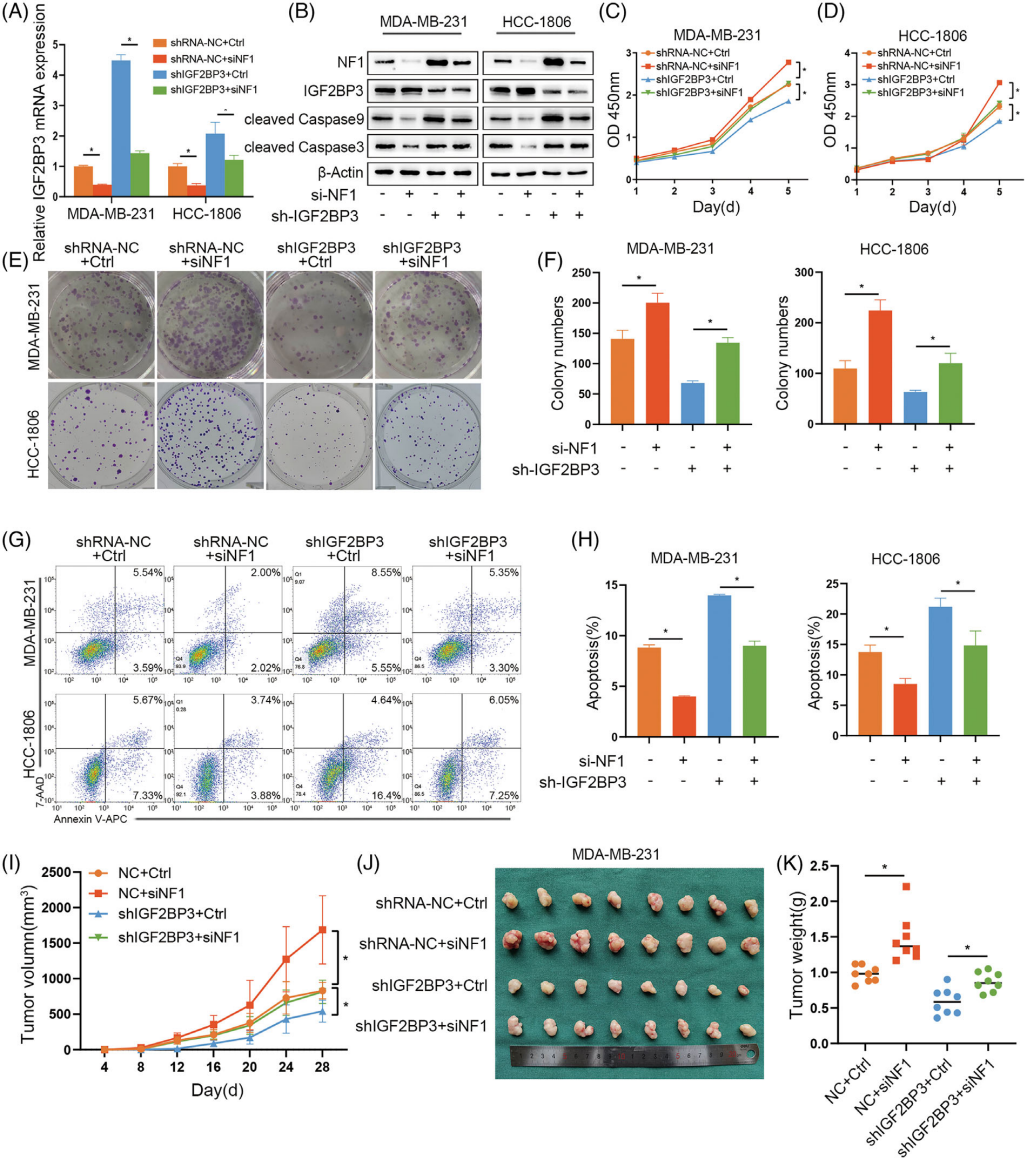
Neurofibromin 1 (NF1) reversed the inhibition of proliferation and promotion of apoptosis induced by IGF2BP3 knockdown. (A and B) IGF2BP3 knockdown and the control groups of MDA‐MB‐231 and HCC‐1806 cells were transfected to knockdown NF1, confirmed by qRT‐PCR and western blotting. (C–F) CCK‐8 assays and colony formation assays were performed to analyse the proliferation ability of MDA‐MB‐231 and HCC‐1806 cells. (G and H) Flow cytometry assays was used to verify the apoptosis analysis of NF1 knockdown in shRNA‐NC and shIGF2BP3 cells. (I–K) Tumour volume and weight in NF1 knockdown and control groups in shRNA‐NC and shIGF2BP3 MDA‐MB‐231 cells in nude mice at different time points. Data are shown as the mean ± SEM; ^*^ *p* < .05.

IGF2BP3 knockdown and the control MDA‐MB‐231 and HCC‐1806 cells were infected with siRNA to suppress NF1 expression. The efficiency of the transfection was validated through qRT‐PCR and western blotting (Figure 7A,B). The CCK‐8 assays and colony formation assays indicated that the NF1 suppression group demonstrated a heightened ability of proliferation in MDA‐MB‐231 and HCC‐1806 cells, while the proliferation ability was decreased by the knockdown of IGF2BP3 (Figure 7C–F). Moreover, in the flow cytometry analysis, the NF1 suppression group could decrease the early and late apoptotic cells in MDA‐MB‐231 and HCC‐1806 cells. In contrast, cell apoptosis was significantly increased by the knockdown of IGF2BP3 (Figure 7G,H). Moreover, downregulation of NF1 increased the expression of cleaved‐caspase 3 and 9 (Figure 7B). In xenograft models, the tumour volume of the NF1 suppress group increased faster than the control group in the shRNA‐NC group or shIGF2BP3 group (Figure 7I,J), and the same result was shown in the control group tumour weights (Figure 7K). These results confirmed that NF1 could reverse the proliferation inhibition and promote the apoptosis conducted by IGF2BP3 knockdown.

## 4 DISCUSSION

m6A is the most abundant reversible modification in eukaryotic mRNA. Due to the development of high‐throughput sequencing technology, researchers can pinpoint the exact m6A site and reveal its function in biological and pathological processes.^40^ More and more evidences indicate that m6A modifications are related to different solid tumours.^41^, ^42^, ^43^ TNBC has a higher probability of metastasis and local recurrence, with the poor survival in patients with TNBC. Recently, epigenetic regulation plays a significant role in the oncogenesis and development of TNBC.^44^ However, there have been few studies on the introduction of m6A and its specific potential effects in TNBC.

IGF2BP3 acts as an oncogenic role and shows significantly high expression in multiple cancers, associated with poor survival.^45^ This study revealed that IGF2BP3 was obviously upregulated in TNBC tissues compared with normal tissues. Moreover, IGF2BP3 was overexpressed in TNBC compared with other subtypes at both the mRNA and protein levels, suggesting that IGF2BP3 might act as a significant oncogene in TNBC. IGF2BP3 is regulated by the genomic alterations, post‐translational modifications and transcriptional control.^46^ According to the previous report, part of pancreatic cancers and thyroid tumours had a specific chromosomal balanced translocation locus on 7p15.3 between the IGF2BP3 chromosomal, resulting in stable overexpression of IGF2BP3.^47^ Moreover, the IGF2BP3 promoter demethylated in CpG islands was a feature of intrahepatic cholangiocarcinoma compared with normal liver tissue.^35^ Furthermore, TCGA also confirmed that the DNA methylation levels of the IGF2BP3 promoter are negatively correlated with IGF2BP3 mRNA expression.^47^ Recent studies have shown that the IGF2BP3 promoter is hypomethylated in TNBC and indicated that the methylation levels in the promoter region are crucial in regulating IGF2BP3 expression. In the mammalian genome, DNA methylation is dynamically regulated by writers (DNMT1/3A/3B) and erasers (TET1/2/3). This study revealed that knockdown of TET3 resulted in the decrease in IGF2BP3 expression and IGF2BP3 promoter luciferase activity. The specific DNA‐methylated sites in the IGF2BP3 promoter were identified by MSP‐PCR. These results were sufficient to display that the upregulation of IGF2BP3 is related to TET3‐mediated promoter hypomethylation in TNBC.

The dysregulation of IGF2BP3 expression in TNBC suggested its potential role in tumourigenesis. For example, IGF2BP3 promoted the proliferation ability by regulating the expression of MYC through mRNA stabilisation in gastric cancer.^48^ It also regulated cell cycle and angiogenesis by binding the m6A‐modified VEGF and CCND1 in colon cancer.^49^ However, there have been few studies in BC via an m6A‐dependent manner by IGF2BP3. Accordingly, our studies indicated that IGF2BP3 knockdown inhibited the proliferation and promoted apoptosis in vitro, whereas IGF2BP3 overexpression displayed the opposite effects. Moreover, IGF2BP3 knockdown decreased the tumourigenesis of TNBC cells in vivo. To clarify the molecular mechanism of how IGF2BP3 promotes expression as an m6A ‘reader’, we performed a multi‐omics analysis by complexing MeRIP‐seq, RIP‐seq and mRNA‐seq. Intersection co‐analysis indicated that IGF2BP3 could recognise m6A methylation to regulate the tumourigenesis process and that NF1 was an essential target of IGF2BP3.

NF1 encodes a GAP that terminates Ras/MAPK signalling pathway by irritating the hydrolysis of Ras•GTP to inactive Ras•GDP resulting in the hyperactivation of Ras and its downstream signalling elements.^50^ Functional studies have shown that NF1 was involved in signalling pathways and responsible for different cellular processes.^51^, ^52^ However, the relationship between NF1 and TNBC has not been reported. IGF2BP3 reads m6A modification by regulating the targeted mRNAs stabilisation.^18^ Correspondingly, we discovered that IGF2BP3 knockdown increased the expression and half‐time of NF1 mRNA and that RNase inhibitors could block the regulation of IGF2BP3 overexpression on NF1 mRNA degradation. These results indicated that NF1 was negatively correlated with IGF2BP3 expression, which showed a poor prognosis in TNBC. Moreover, NF1 was significant for the proliferation and apoptosis of TNBC cells. Moreover, knockdown of NF1 rescued the phenotypes of IGF2BP3 knockdown cells in vivo and in vitro, elucidating the significance of NF1 in TNBC development. These findings indicated that IGF2BP3 could decrease NF1 stabilisation via an m6A‐dependent manner, leading to TNBC development.

## 5 CONCLUSIONS

In summary, our study demonstrated that TET3‐mediated IGF2BP3 promoter hypomethylation leads to the upregulation of IGF2BP3 in TNBC. Moreover, IGF2BP3 recognised the m6A target on NF1 mRNA and decreased the stabilisation of NF1. IGF2BP3 decreased NF1 expression via an m6A‐dependent manner as an oncogenic regulator.

**FIGURE 8:**
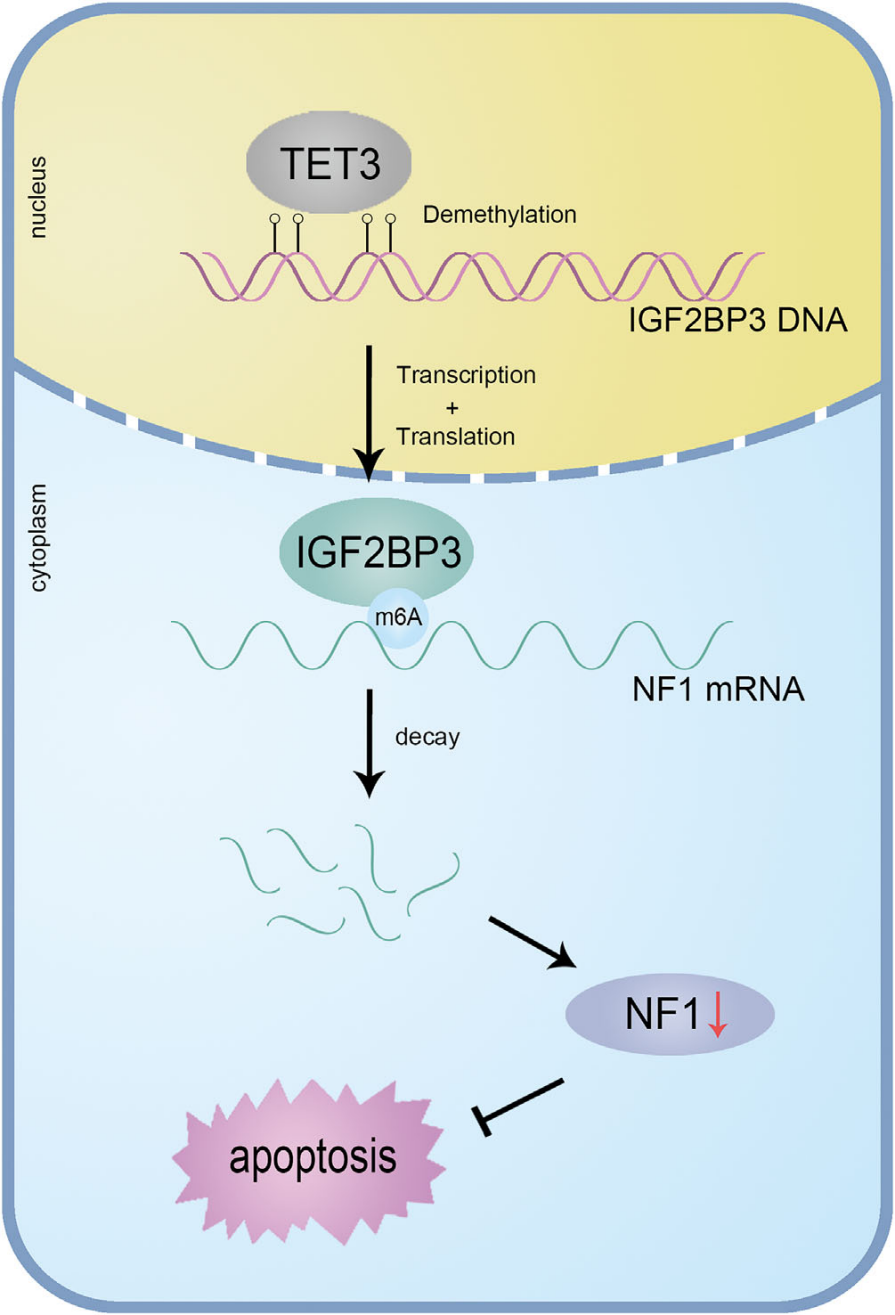
Graphic illustration of IGF2BP3 modulating triple‐negative breast cancer (TNBC) proliferation and apoptosis via decreasing neurofibromin 1 (NF1) mRNA stability in an N6‐methyladenosine (m6A)‐dependent manner.

Accordingly, we found a novel mechanism of the IGF2BP3–NF1 axis in regulating TNBC proliferation, which may be investigated for TNBC prognosis, diagnosis or treatment (Figures 8).

## CONFLICT OF INTEREST STATEMENT

The authors declare they have no conflicts of interest.

## Supporting information

Supporting Information

Supporting Information

Supporting Information

Supporting Information

Supporting Information

## Data Availability

The data that support this study are available upon reasonable request from the corresponding author.
