# Supplementary material for: IGF2BP3 mediates the mRNA degradation of NF1 to promote triple‐negative breast cancer progression via an m6A‐dependent manner
Source: Clin Transl Med. 2023 Sep 24;13(9):e1427. doi: 10.1002/ctm2.1427 (PMC10518495; doi:10.1002/ctm2.1427)
Supplement: Supplementary file 1 — Supporting Information [file CTM2-13-e1427-s001.docx]

**Figure S1**

**
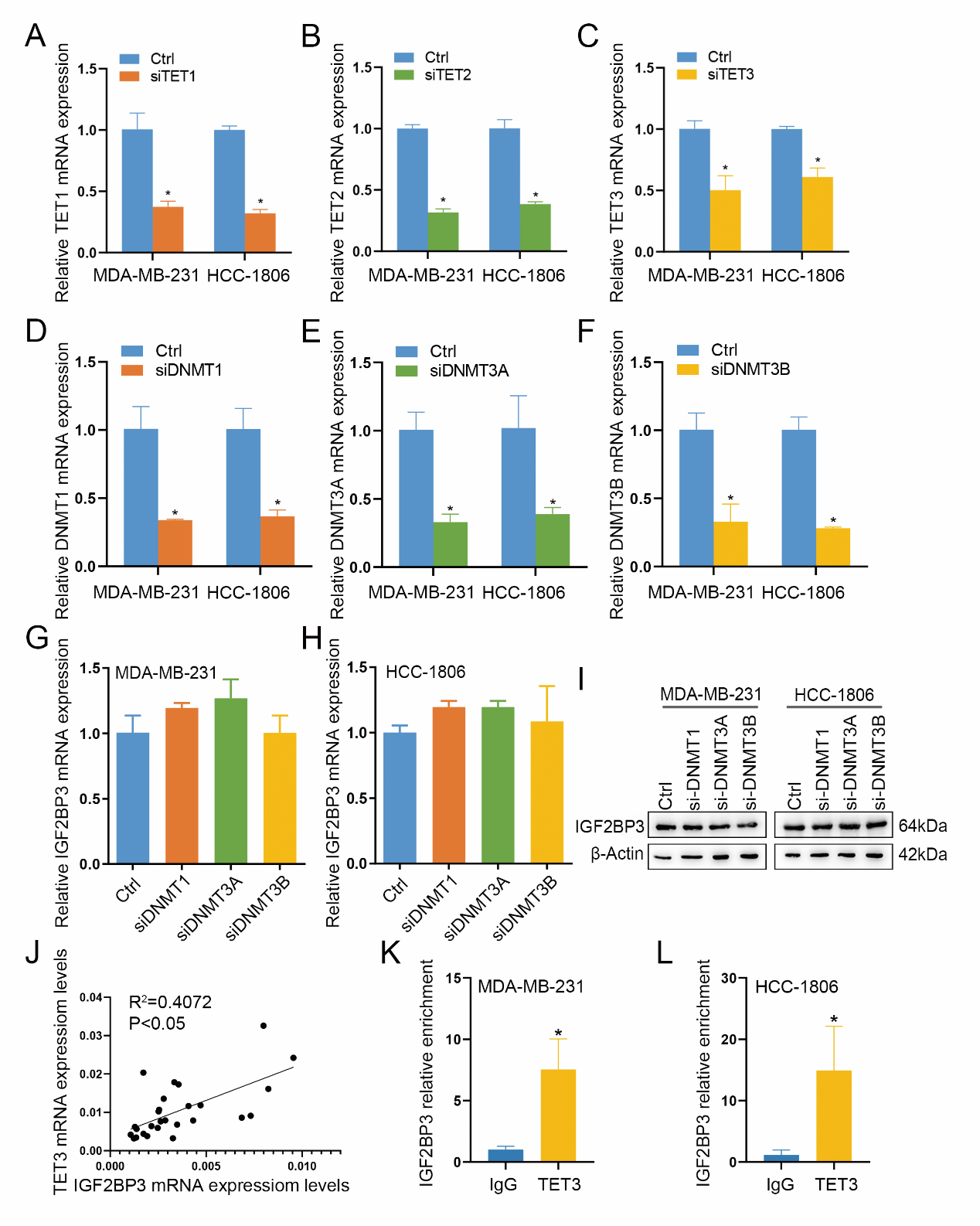
**

**Figure S2**

**
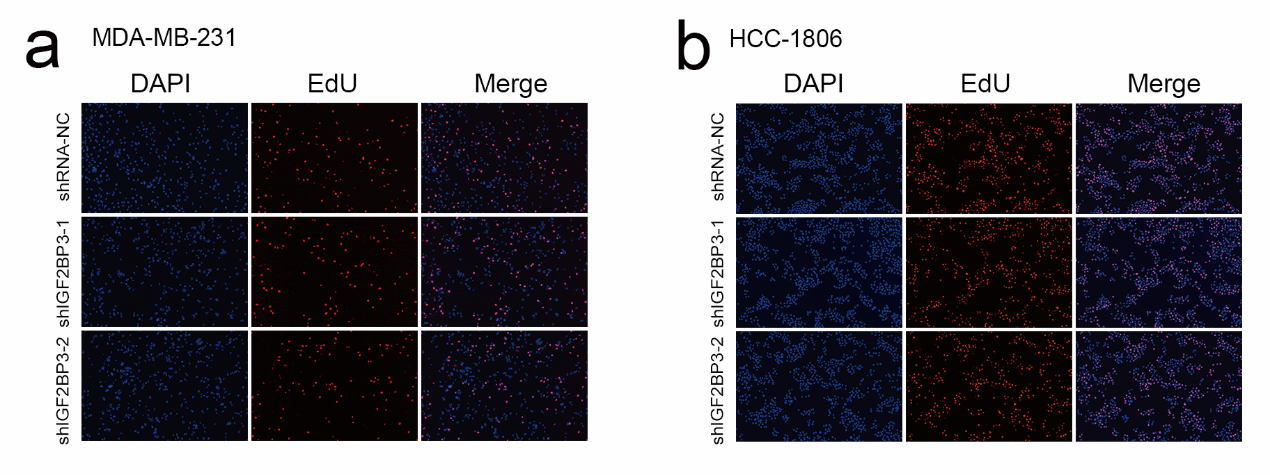
**

**Figure S3**

**
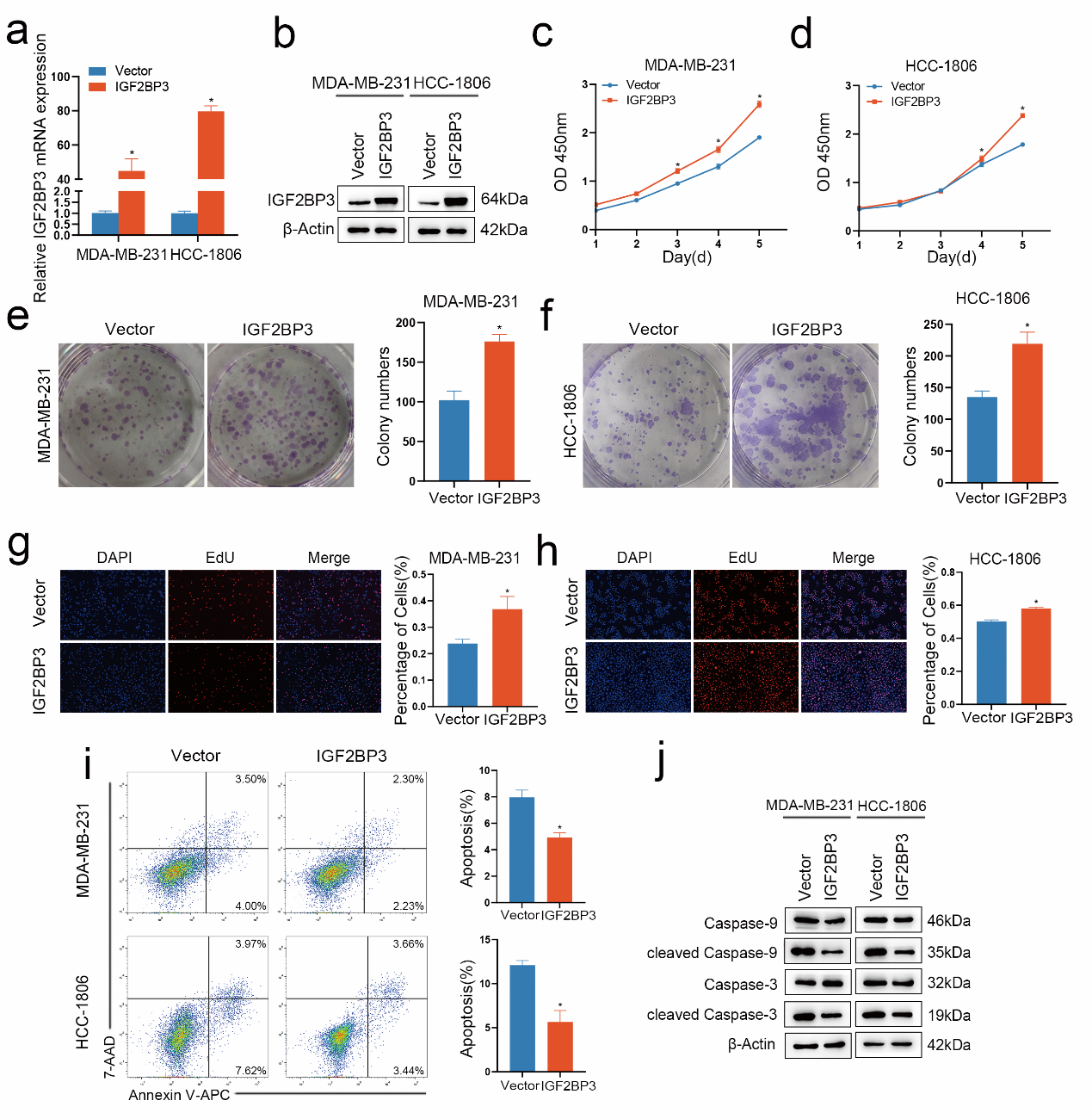
**

**Figure S4**

**
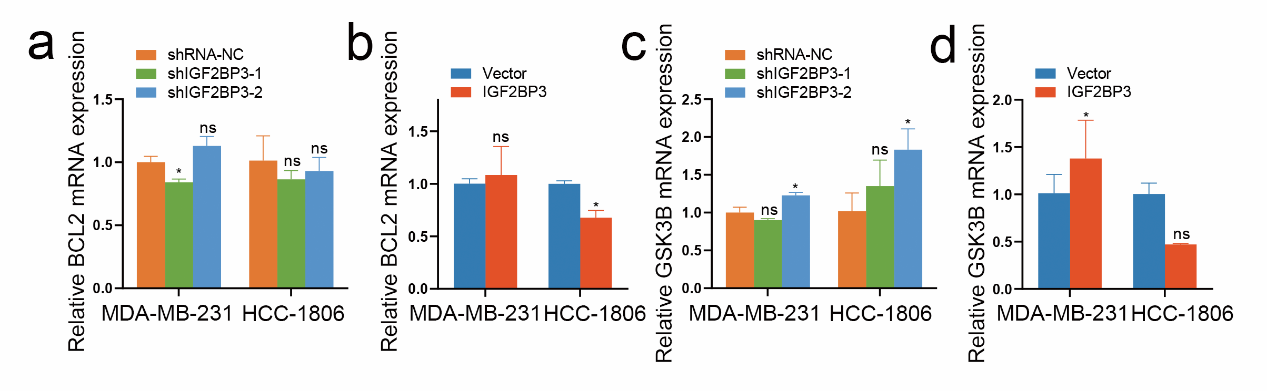
**

**Figure Legends**

**Figure S1：Efficiency of transfection with small interference and IGF2BP3 promoter enrichment by ChIP-qPCR**

**a-f** MDA-MB-231 and HCC-1806 cell lines using specific small interfering RNAs (siDNMT1/3A/3B, TET1/2/3) and qRT-PCR were applied to verify transfection efficiency. Data were shown as mean ± SEM, *p <0.05. **g-h** qRT-PCR was used to confirm the IGF2BP3 expression at mRNA levels. **i** Western blot was used to confirm the IGF2BP3 expression at protein levels. Data were shown as mean ± SEM, *p<0.05. **j** Correlation analysis between IGF2BP3 and TET3 mRNA expression in TNBC tissues (n = 27). **k-l** IGF2BP3 promoter enrichment for TET3 was detected by ChIP-qPCR in MDA-MB-231 and HCC-1806 cell lines. Data were shown as mean ± SEM, *p <0.05.

**Figure S2：Edu assays after knockdown of IGF2BP3**

**a-b** The proliferation ability after knockdown of IGF2BP3 was evaluated by Edu assays in MDA-MB-231 and HCC-1806 cell lines.

**Figure S3: Overexpression of IGF2BP3 promoted proliferation and inhibited the apoptosis of TNBC in *vitro***

**a-b** MDA-MB-231 and HCC-1806 cell lines were transfected with lentivirus (IGF2BP3) to overexpress IGF2BP3 expression. qRT-PCR **(a)** and Western blot **(b)** were applied to verify transfection efficiency. **c-d** The proliferation ability after overexpressing IGF2BP3 was evaluated by CCK8 assay in MDA-MB-231 and HCC-1806 cell lines. **e-f** The proliferation ability after overexpressing IGF2BP3 was evaluated by a colony formation assay in MDA-MB-231 and HCC-1806 cell lines. **g-h** The proliferation ability after overexpressing IGF2BP3 was evaluated by Edu assays in MDA-MB-231 and HCC-1806 cell lines. **i-j** Flow cytometry assay and western blot assay were used to confirm the apoptosis analysis induced by overexpression of IGF2BP3. Data were shown as mean ± SEM, *p <0.05.

**Figure S4:** **Validate the candidate genes**

**a-d** qRT‒PCR was performed in MDA-MB-231 and HCC-1806 cell lines with IGF2BP3 knockdown or overexpression to validate the candidate genes. Data were shown as mean ± SEM, *p <0.05
