# Supplementary material for: IGF2BP3 mediates the mRNA degradation of NF1 to promote triple‐negative breast cancer progression via an m6A‐dependent manner
Source: Clin Transl Med. 2023 Sep 24;13(9):e1427. doi: 10.1002/ctm2.1427 (PMC10518495; doi:10.1002/ctm2.1427)
Supplement: Supplementary file 2 — Supporting Information [file CTM2-13-e1427-s004.docx]

**Table S1.  Lentivirus and siRNA sequences.**

| Name | Sequences (5’-3’) |
| --- | --- |
| shIGF2BP3-1 | GGTGAAACTTGAAGCTCAT |
| shIGF2BP3-2 | CCAGACACCTGATGAGAAT |
| si-NF1  si-TET1 | CCACUCCCUACUGAAUAAATT  GCAAGACACCCAAGUCCUUTT |
| si-TET2 | GGCAGUGCUAAUGCCUAAUTT |
| si-TET3 | GGAAAUAAAGGCUGGUGAATT’ |
