# Supplementary material for: IGF2BP3 mediates the mRNA degradation of NF1 to promote triple‐negative breast cancer progression via an m6A‐dependent manner
Source: Clin Transl Med. 2023 Sep 24;13(9):e1427. doi: 10.1002/ctm2.1427 (PMC10518495; doi:10.1002/ctm2.1427)
Supplement: Supplementary file 3 — Supporting Information [file CTM2-13-e1427-s005.docx]

**Table S2. Oligonucleotide sequences used in this study**

| Primes |  | Sequences (5’-3’) |
| --- | --- | --- |
| IGF2BP3 | Forward | TTGCAGGAATTGACGCTGTA |
|  | Reverse | ACCCAAGGCGTTCAGATTTA |
| TET1 | Forward | TGCACGCTACCACTCCTGT |
|  | Reverse | TCAGAGGACTGTTCTGGACCT |
| TET2 | Forward | TTGCTAAGTGGGTGGTTCG |
|  | Reverse | GGTGAGCGTGCCGTATTT |
| TET3 | Forward | TGCGTCGAACAAATAGTGGA |
|  | Reverse | CTGCGGATCACCCACTTTG |
| NF1 | Forward | AGGTCCGCTCTCCCTTAGA |
|  | Reverse | TAACTGGAACGGAAGGCAAT |
| β-actin | Forward | TCACCCACACTGTGCCCATCTACGA |
| For methylated (M)  For unmethylated (U) | Reverse  Forward  Reverse  Forward  Reverse | CAGCGGAACCGCTCATTGCCAATGG  ATTAATGGTAAACGCGAAGTC  AACAAAACAAACGAACGACG  ATTAATGGTAAATGTGAAGTTGAGA  AAATAAAAACAAAACAAACAAACAACAC |
