# Supplementary material for: IGF2BP3 mediates the mRNA degradation of NF1 to promote triple‐negative breast cancer progression via an m6A‐dependent manner
Source: Clin Transl Med. 2023 Sep 24;13(9):e1427. doi: 10.1002/ctm2.1427 (PMC10518495; doi:10.1002/ctm2.1427)
Supplement: Supplementary file 4 — Supporting Information [file CTM2-13-e1427-s002.docx]

**Table S3. Sequence of luciferase vector**

| luciferase vector | Sequence |
| --- | --- |
| A | GGGGTGTCATGGCGGCGTCTCGGACTGTGATGGCTGTGGGGAGACGGCGCTAGTGGGGAGAGCGACCAAGAGGCCCCCTCCCCTCCCCGGGTCCCCTTCCCCTATCCCCCTCCCCCCAGCCTCCTTGCCAACGCCCCCTTTCCCTCTCCCCCT |
| B | AGCCATATGAAATTGTAGTGGACCTTACCCATACCGGGCCTAGCAATCGCTTTAAAACAGACTTTCTCTCTAAGTGGTTTGTTGTTTTTCCTGGCTTTGCTTACGACAACGTCTCCGCAGTCTATATCTATAACTGTAACTCCTGGGTCAGGGAGTACACCAAGTATCATGAGCGGCTGCTGACTGGCCTCAAAGGTAGCAAAAGGCTTGTTTTCATAGACTGTCCTGGGAAACTGGCTGAGCACATAGAGCATGAACAACAGAAACTACCTGCTGCCACCTTGGCTTTAGAAGAGGACCTGAAGGTATTCCACAATGCTCTCAAGCTAGCTCACAAAGACACCAAAGTTTCTATTAAA |
| C | TTTACATACACTACTAACTCTGGTTAACAAACACAGAAATTGTGACAAATTTGAAGTGAATACACAGAGCGTGGCCTACTTAGCAGCTTTACTTACAGTGTCTGAAGAAGTTCGAAGTCGCTGCAGCCTAAAACATAGAAAGTCACTTCTTCTTACTGATATTTCAATG |
| D | AGTATTATTAATGAGTTTACCATAGAATTGTTGGAAATACTGAAGACAGGTGCAATTTACTAAACTTTTGTTTTTAAACTATTGTAGAGGCTGCATTAGAAGAAAATGTTTATAATGACAGAGCAACTATGACTATATAAAAAAGCTGAAATTAGAACTGTGTTTAGAAATAGATCAGTAACCCAGTGCCAAGGATGCCAAGCTGCCACCATGGTCTTGGCTCTCCCACAACCCAGTGTTTCTGGGGTAAGTTTCACAGTTTCTAGGCCCTGGAATAGCAGGCAGTGTAAGCCTTTGATAACTTTAGTTCGATGTTTTTCTTGTTTTTGTTTGTTGGTTTGGTGCATATGATAGTGGGTGTTATGCTATTTTGCTCTTCCCATCAAAATAAAGAAACTTCCAGAGGTTTACTGT |
| E | AGGAGTTTGAAAACTTAACTAAGGTTTAAAATTTACCTTGTTTAAAGAACTTCTGACTTTTGAGGAAAATCTAGCTTTCCAAGTAACTAAAATGTACATGAGATAAACCTCTCACCACTATGTGTCCCTTGAGAAATGCAACACTTTTTTAGTCTTCATACTTGTAATCTATAAAAGAAATTCTGAAGTTTAGACCAAGTTGCCCATTTCTGCGTAATTGACATAAGTTCTGTTAAAAATATTATAAGTAATTCGTTTCGGTTTGTAGATGTTTCCCCTGACTTGTTAAAGAGGAAACCAGGAACTCAGTCATGTTTTTGTCCTGGATAATCTACCTGTTATGCCAGTACTCCCATCCGAGGGGCATGCCCTTAGTTGCCCAGATGGAGATGCAGTTCAGTAGATTTGGGGCAAAGTGGCTACAGCTCTGTCTTCCATTCACTCAACACCTGTTCATGACTGAGCCAGGTGCCCAGGACACATCCTAAACAGTCAGCT |
| B-mut | AGCCATATGAAATTGTAGTGGACCTTACCCATACCGGGCCTAGCAATCGCTTTATTCTCTCTAAGTGGTTTGTTGTTTTTCCTGGCTTTGCTTACGACAACGTCTCCGCAGTCTATATCTATAACTGTAACTCCTGGGTCAGGGAGTACACCAAGTATCATGAGCGGCTGCTGACTGGCCTCAAAGGTAGCAAAAGGCTTGTTTTCATGTCCTGGGAAACTGGCTGAGCACATAGAGCATGAACAACAGAAACTACCTGCTGCCACCTTGGCTTTAGAAGAGGACCTGAAGGTATTCCACAATGCTCTCAAGCTAGCTCACAAAGACACCAAAGTTTCTATTAAA |
| C-mut | TTTACATACACTACTAACTCTGGTTACAGAAATTGTGACAAATTTGAAGTGAATACACAGAGCGTGGCCTACTTAGCAGCTTTACTTACAGTGTCTGAAGAAGTTCGAAGTCGCTGCAGCCTAAAACATAGAAAGTCACTTCTTCTTACTGATATTTCAATG |
